# B-cell dynamics during experimental endotoxemia in humans

**DOI:** 10.1042/BSR20182347

**Published:** 2019-05-17

**Authors:** Alexandra Brinkhoff, Ye Zeng, Annette Sieberichs, Sebastian Dolff, Xu Shilei, Ming Sun, Harald Engler, Sven Benson, Johannes Korth, Manfred Schedlowski, Andreas Kribben, Oliver Witzke, Benjamin Wilde

**Affiliations:** 1 Department of Nephrology, University Hospital Essen, University of Duisburg-Essen, Essen, Germany; 2 Institute of Medical Psychology and Behavioral Immunobiology, University Hospital Essen, University of Duisburg-Essen, Essen, Germany; 3 Department of Infectious Diseases, University Hospital Essen, University of Duisburg-Essen, Essen, Germany

**Keywords:** B-cells, BAFF, Endotoxemia, inflammation, LPS, sepsis

## Abstract

Recently, B cells with regulatory functions suppressing T-cell immunity were identified. Inflammation in the context of sepsis is characterized by a profound immune dysfunction increasing the patient’s risk for additional infections. The impact of endotoxemia on B-cell dynamics, regulatory B cells (Breg) and its contribution to immune dysfunction is unknown. It is the aim of the present study to characterize the dynamics of the B-cell compartment and Breg in an experimental human endotoxemia model.

In this randomized placebo-controlled cross-over study, 20 healthy males received an intravenous injection of endotoxin (*Escherichia coli* lipopolysaccharide, LPS, 0.8 ng/kg body weight) or placebo (saline 0.9%) on two otherwise identical study days. B cells were analyzed by flow cytometry at baseline and repeatedly up to 72 h after endotoxin/placebo injection.

Absolute CD19^+^ B cells counts showed a significant decrease 3 h after endotoxin injection. Memory B cells were partially depleted from the circulation; the total number of Breg was significantly diminished 3 h after LPS challenge. Production of anti-inflammatory interleukin (IL)-10 (IL-10) by Breg was unaltered after LPS challenge. Systemic B-cell activating factor (BAFF) levels were significantly increased with a maximum after 24 h and remained increased up to 72 h post-injection.

Endotoxemia causes a transient depletion of memory B cells and Breg from the circulation. However, the functional capacity of B cells to produce IL-10 is not impaired.

## Introduction

Sepsis is an immune dysfunction affecting both pro- and anti-inflammatory mechanisms [1]. The initial pro-inflammatory phase is characterized by an inappropriate immune response to a host pathogen followed by an anti-inflammatory phase rendering the host susceptible to secondary infections. Failure to clear and control these secondary infections is a major contributor to lethal outcome of sepsis [2]. The sequence and mechanisms of the anti-inflammatory mechanisms have not been unraveled entirely. However, it has been consistently shown that effector responses are severely compromised during this anti-inflammatory phase [3,4]. The impact of sepsis/systemic inflammation on B cells is not clear. Under physiological circumstances, activated effector B cells may differentiate into plasma cells or memory B cells driving the humoral immune response and act as antigen-presenting cells to activate effector T cells (Teff). Septic patients show a disturbed B cell-mediated immunity [5–8]. In addition, Leentjens et al. [9] demonstrated a transient reduction in circulating B cells in human endotoxemia model. Only recently, an additional subset of B cells with regulatory properties (Breg) has been described with this subset being a potent inhibitor of Teff responses limiting pro-inflammatory immune responses [10,11]. Breg may physiologically limit the effector immune response during infections of the host to tailor the strength of the response to the level needed. In addition, Breg are important contributors to immune tolerance and Breg dysfunctions have been described in autoimmune diseases and malignancies [12–17]. Some evidence indicates that specific regulatory T-cell populations mediate sepsis-induced immune dysfunction [17]. Like Tregs, Breg bear potent immunosuppressive capacity. Thus, it is conceivable that Breg may have a role in sepsis-induced immune dysfunction. However, data on human B cells in sepsis are currently inconsistent which is probably due to an inherent bias of such patient studies [5–8]. Thus, to further analyze the impact of systemic inflammation on the B-cell compartment, we took advantage of the human experimental endotoxemia model. The endotoxemia model is used as an established experimental approach to assess the effects of sepsis-like systemic inflammation and endotoxemia [4,18–20]. Therefore, the aim of the present study was: (i) to investigate the abundance of circulating B-cell subsets during endotoxemia in healthy volunteers and (ii) to study in which way endotoxemia impacts Breg.

## Materials and methods

### Participants

This cross-over study is a placebo-controlled, randomized and single-blinded trial as previously reported [18,19,21]. Twenty healthy men were included for analyzing clinical and immunological parameters. The participants were recruited by public advertisement. The screening and safety procedure provided a personal interview under medical supervision, a physical examination including an assessment of blood and clinical chemistry parameters. Laboratory screening was conducted before each study day (lipopolysaccharide (LPS) vs. placebo) and up to 1 week after completing the study. Participants were excluded with reported or current medical conditions, body mass index (BMI) < 19.0 or ≥ 27.0 kg/m^2^, current medication, smoking, regular and/or high alcohol consumption, present allergies, vaccinations within the last 2 months and with reported extensive sport exercises 24 h before and after the study days. One participant did not complete the +72 h time point within the LPS condition due to a case of family-related acute gastroenteritis. The study protocol was approved by the local ethics committee of the University Hospital Essen, Germany (permission sign: 15-6533-BO). All volunteers provided written consent and received financial compensation for study participation.

### Study protocol

The study protocol has been previously described in detail [21]. Briefly, participants received either LPS (LPS condition) or placebo (placebo condition) on two otherwise identical study days in a randomized order, with a minimum of 7 days between study conditions. On both study days, an intravenous catheter was inserted in a cubital vein after arrival at the lab for repeated blood drawing and endotoxin injection. After a 30-min resting period, heart and breathing rate, pulse oximetry (Kernmed Oled, Ettlingen, Germany) and blood pressure (Dinamap Compact T, Critikon, Norderstedt, Germany) were assessed. One hour after arrival, subjects received an intravenous injection of either LPS (0.8 ng/kg body weight *Eschericha coli* LPS, 200 ng/ml, LOT HOK354, USP The United States Pharmacopeial Convention, Inc., Rockville, MD, U.S.A.) or placebo (saline 0.9%) under continuous vital sign monitoring. The LPS charge had been consigned to the German Federal Agency for Sera and Vaccination (Paul-Ehrlich Institute, Langen, Germany) for microbial safety testing, and was stored in endotoxin-free borosilicate tubes (Pyroquant Diagnostik, Mörfelden-Walldorf, Germany) at −20°C until use. Blood samples were collected before and up to +72 h after LPS or placebo injection (see below). Following each blood sampling, vital signs were assessed under supervision of an internal physician.

### Samples

EDTA anticoagulated peripheral blood (PB) samples for soluble cytokine analysis were collected in blood collection tubes (Sarstedt, Nümbrecht, Germany) at baseline (= before saline or LPS injection), +1, +2, +3, +4, +6, +24, +48 and +72 h after injection of saline or LPS. PB mononuclear cells (PBMCs) collection was performed in heparin-coated tubes at baseline, +3, +24, +48 and +72 h after LPS/placebo injection. EDTA and heparin plasma was separated by centrifugation and stored at −80°C. PB samples were prepared and cultured *ex vivo* with a maximum period of 1 h after collection.

### PBMC isolation

PBMCs were isolated by Ficoll density gradient centrifugation. The gradient was a commercially available Ficoll separation medium (Lymphoprep, Stem Cell Technologies, Cologne, Germany). PBMCs were resuspended in RPMI 1640 medium with Glutamax (Gibco Life Technologies, Darmstadt, Germany) supplemented with 10% heat-inactivated fetal calf serum (Greiner Bio-One, Frickenhausen, Germany), of penicillin (100 U/ml) and streptomycin (1 µg/ml) as well as non-essential amino acids (NEAs) (diluted according to manufacturer’s instructions, MEM NEA) and sodium pyruvate (1 mM, all from Gibco Life Technologies, Schwerte, Germany).

### Flow cytometry and cell stimulation

The fluorescence–conjugated antibodies were supplied by BD Biosciences (BD Biosciences, Erembodegen, Belgium), eBioscience (Ebioscience, Schwerte, Germany), Biolegend (Biolegend, London, United Kingdom), Invitrogen (Invitrogen, Schwerte, Germany) and Beckman Coulter (Beckman Coulter, Krefeld, Germany). The following monoclonal antibodies were used for the Breg surface phenotyping: anti-CD19/Pacific Blue (PcB), anti-CD24/Phycoerythrin-Cyanin-7 (PE-CY7), anti-CD38/fluorescein isothiocyanate (FITC), anti-CD268 (BAFF-R)/allophycocyanin (APC) and anti-CD274 (PDL-1)/PE. Surface phenotyping of B cells and analysis of cytokine-producing Breg were performed with freshly isolated PBMCs as previously described [10,22]. Breg were phenotypically defined via surface staining as CD19**^+^**CD24**^hi^**CD38**^hi^** according to Blair et al. [10]. Further B-cell subsets were defined based on CD24 and CD38 expression pattern according to Carsetti et al. [23]: mature naïve B cells were defined as CD19**^+^**CD24**^lo^**CD38**^lo^**, plasmablasts as CD19**^+^**CD24**^lo^**CD38**^hi^** and memory B cells were identified as being CD19**^+^**CD24**^hi^**CD38**^lo^**. For surface staining, PBMCs were incubated with antibodies for 30 min and analyzed immediately after washing with Dulbecco’s phosphate-buffered saline (DPBS 1×, Gibco, Life Technologies, Darmstadt, Deutschland). Isotype controls were used to confirm specificity of staining and to discriminate background staining.

**Figure 1:**
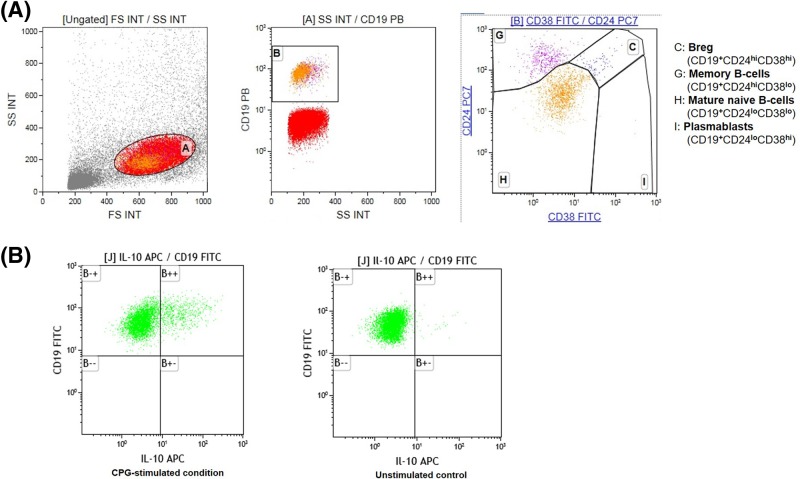
Representative flow cytometric data (**A**) Gating strategy to identify memory B cells, plasmablasts, mature naïve B cells and CD24^hi^CD38^hi^ Breg. (**B**) Representative dot plots of IL-10^+^ Breg and the corresponding control; plots are gated on viable, activated, singlet CD19^+^ B cells. IL-10 production was assessed by flow cytometry after culture in presence or absence (control condition) of TLR9 agonists for 72 h and subsequent restimulation with PMA/ionomycin for 6 h.

Cytokine-producing Breg were defined as CD19**^+^**7AAD**^neg^**CD69**^+^**IL-10**^+^** B cells (Figure 1). To assess cytokine-producing Breg, PBMCs were first divided into a stimulated and unstimulated condition. For analysis of cytokine responses, PBMCs were cultured in presence or absence of CPG-ODN 2006 (0.1 µM, InvivoGen, Toulouse, France) at 37°C, 5% CO_2_ for 72 h as described prevsiously [22]. Thereafter, PBMCs were restimulated with PMA (10 ng/ml, Sigma–Aldrich, Taufkirchen, Germany), Ionomycin (1 µg/ml, Sigma–Aldrich) in the presence of Brefeldin A (5 µg/ml, BD Biosciences) for 6 h followed by surface staining, fixation and permeabilization (CytoFix/CytoPerm kit, BD Biosciences, Erembodegen, Belgium).

For intracellular flow cytometric analysis the Breg staining was performed with: anti-CD3/Horv450, anti-CD19/PB, anti-7AAD/peridinin–chlorophyll–protein complex (PerCP) and interleukin (IL) 10 (IL-10)/APC. After fixation and permeabilization, PBMCs were stained intracellularly for IL-10 (APC) and CD69 (PE-CY7). Appropriate isotype controls were used to confirm specificity of staining and to discriminate background staining. CD69 expression was used as a control for the activation procedure and 7AAD served as a viability control. Doublets were excluded by gating scatter-height (forward scatter height (FSC-H)) versus forward-scatter area (FSC-A) [22]. Data were acquired on a 3-laser Navios flow cytometer (Beckman Coulter, Krefeld, Germany), equipped to detect ten fluorescent parameters. Compensation and data analysis were done with Kaluza Analysis 1.5a software (Beckman Coulter, Krefeld, Germany).

### Cytokine analyses

Plasma concentrations of B-cell activating factor (BAFF), IL-10 and TNFa were measured by enzyme-linked immunosorbent assay (ELISA) (Human Quantikine ELISA, R&D Systems, Wiesbaden-Nordenstadt, Germany) according to the manufacturer’s instructions using a Fluostar Optima microplate reader (BMG Labtech, Offenburg, Germany).

### Statistical analyses

Data are shown as mean and standard error of the mean (SEM). Prior to analysis, normal distribution of data was tested using Kolmogorov–Smirnov test. Repeated measures (e.g., cytokine concentrations) were compared between LPS and placebo conditions using two-way repeated measure analysis of variance (ANOVA) with the repeated factors time and condition (i.e., LPS, placebo). All *P*-values on ANOVA analysis indicate ANOVA time × condition interaction. To compare single measurement points separately between LPS and placebo conditions, post-hoc paired *t* tests were calculated applying Bonferroni-correction for multiple testing. Pearson’s r was calculated to analyze correlations between variables. *P*-values <0.05 were considered to be statistically significant. Data analysis was conducted with SPSS 22.0, SPSS Inc. Chicago, U.S.A., and graphs were created using GraphPad Prism® 6 (version 6.01, GraphPad Software, Inc., La Jolla, CA, U.S.A.).

## Results

### Demographic and clinical characteristics of the study cohort

This cross-over study included *n*=20 healthy males of Caucasian ethnicity. Mean age was 26.1 ± 0.9 years (range 18.8–34.9 years), and mean BMI at screening day was 24.2 ± 0.5 kg/m^2^ (range: 19.3–26.9 kg/m^2^). LPS administration led in all participants to the expected changes in vital parameters indicating a systemic immune activation, including transient increases in heart rate, respiratory rate, body temperature and a decrease in systolic blood pressure (Supplementary Figure S1).

### B cell and CD24^hi^CD38^hi^ Breg dynamics during endotoxemia

**Figure 2:**
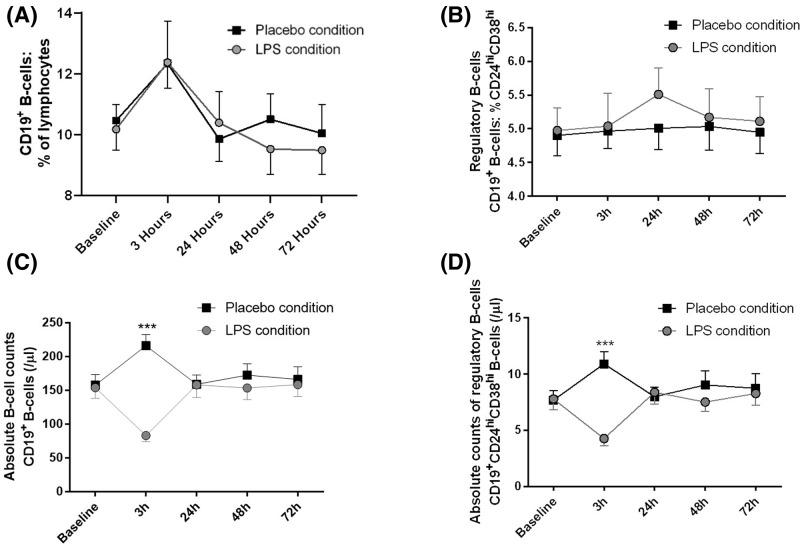
B-cell dynamics after LPS administration (**A**) Relative B-cell numbers, (**B**) relative Breg counts, (**C**) absolute B-cell counts and (**D**) absolute Breg counts were measured up to 72 h after the injection of 0.8 ng/kg body weight LPS- (gray) or placebo (black) (*n*=20). Data are shown as mean ± SEM. Two-way repeated measure ANOVA were performed followed by post-hoc Bonferroni-corrected paired *t* tests. ***P<0.001 represent *P*-values of post-hoc Bonferroni-corrected *t* tests. For results of ANOVA, see text.

Lymphocyte counts decreased significantly after LPS challenge from 1.56 ± X ^∧^10^9^/l at baseline to 0.68 ± X ^∧^10^9^/l after 3 h. Furthermore, T-cell counts also decreased over time and recovered within 24 h (CD3^+^ T cells: baseline 0.92 ± 0.28 ^∧^10^9^/l, 3 h 0.4 ± 0.14 ^∧^10^9^/l, 24 h 0.89 ± 0.29 ^∧^10^9^/l; CD4^+^ T cells: baseline 0.55 ± 0.19 ^∧^10^9^/l, 3 h 0.27 ± 0.09 ^∧^10^9^/l, 24 h 0.55 ± 0.22 ^∧^10^9^/l; CD8^+^ T cells: baseline 0.27 ± 0.1 ^∧^10^9^/l, 3 h 0.11 ± 0.04 ^∧^10^9^/l, 24 h 0.24 ±0.1 ^∧^10^9^/l). Absolute CD19^+^ B cell (Figure 2, F = 23.79, *P*≤0.0001) and CD24**^hi^**CD38**^hi^** Breg counts (Figure 2, F = 16.39, *P*=0.0001) were significantly reduced at 3 h after LPS injection compared with the placebo injection. The relative fraction of CD24**^hi^**CD38**^hi^** Breg (Figure 2: F = 0.324, *P*=0.86) as well as BAFF-receptor and PDL-1 surface expression on CD24**^hi^**CD38**^hi^** Breg did not significantly change over time (BAFF-R: F = 0.10, *P*=0.98; PDL-1: F = 0.64, *P*=0.64, data not shown).

**Figure 3:**
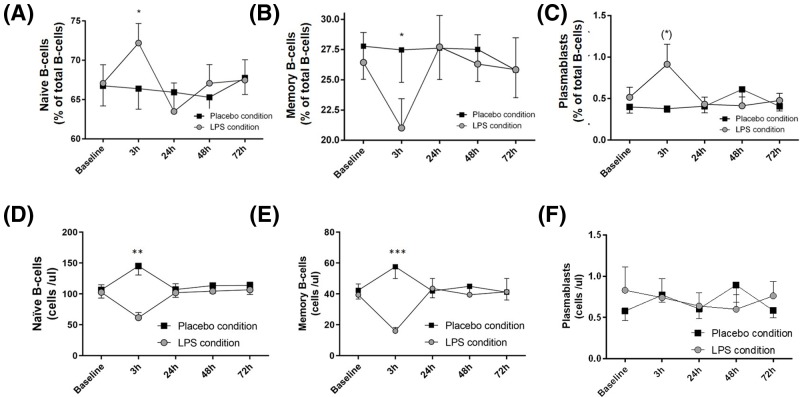
B-cell subpopulations show a transient skewing after LPS challenge (**A**) Naïve B cells showed an increase in relative numbers at 3 h after LPS challenge, whereas (**B**) memory B cells declined. Relative numbers normalized after 24 h and did not differ from the placebo condition. (**C**) Plasmablasts increased 3 h after LPS application and normalized at 24 h. Absolute counts are given for the same subsets (**D**–**F**). Mean ± SEM are given for each time-point measured over 72 h under LPS- (gray) and placebo condition (black); *n*=20. Two-way repeated measure ANOVA analyses were performed followed by post-hoc Bonferroni-corrected paired *t* tests. *P<0.05, **P<0.01, ***P<0.001, results of post-hoc Bonferroni-corrected *t* tests. For results of ANOVA, see text. (*) *P*<0.05, results from post-hoc *t* test, remain non-significant after Bonferroni correction.

LPS challenge induced a transient skewing within the B-cell compartment. The relative fraction of the mature naïve B-cell compartment (Figure 3A, F = 2.69, *P*=0.038) showed a transient increase at 3 h after LPS challenge, whereas the relative fraction of memory B cells (Figure 3B, F = 2.79, *P*=0.032) showed a decrease at 3 h after LPS application. Plasmablasts showed a slight but significant increase at 3 h after LPS administration (Figure 3C, F = 3.88, *P*=0.006; all *P*-values indicate ANOVA time × condition interaction). Absolute counts for the same subsets are depicted in (Figure 3 D-F).

### Functional assessment of Breg during endotoxemia

**Figure 4:**
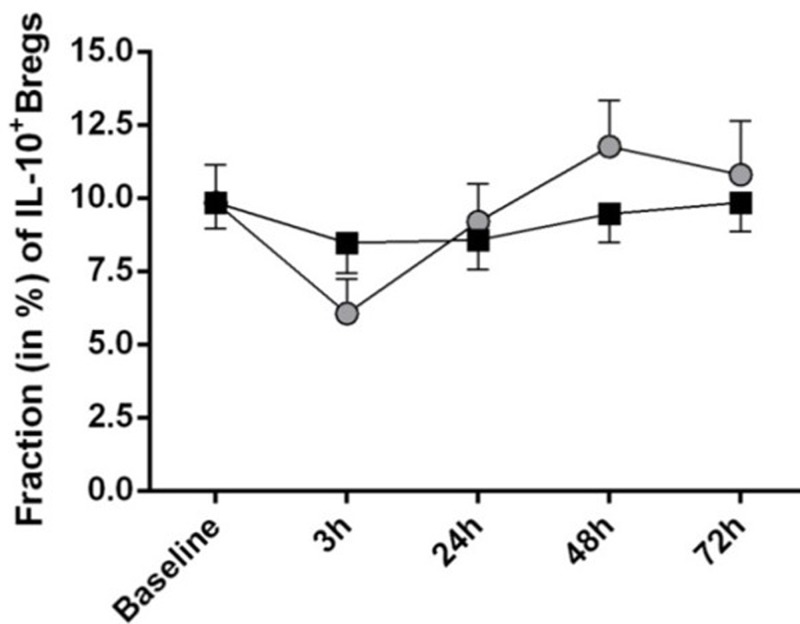
B-cell derived IL-10 production is not altered during endotoxemia The fraction of IL-10 producing B cells was stable over 72 h in both conditions. There was no difference between the LPS and placebo conditions. Data are given as mean ± SEM. Post-hoc Bonferroni-corrected *t* tests were applied.

Besides a transitional phenotype, Breg are characterized by expression of IL-10. Therefore, the intracellular cytokine profile of *ex vivo* stimulated B cells was assessed at five different time points (Figures 1 and 4). There were no significant differences between the LPS and placebo condition with respect to IL-10 producing Breg (F = 1.6, *P*=0.18, *n*=18, ANOVA interaction effects).

### Systemic BAFF levels increase during endotoxemia

**Figure 5:**
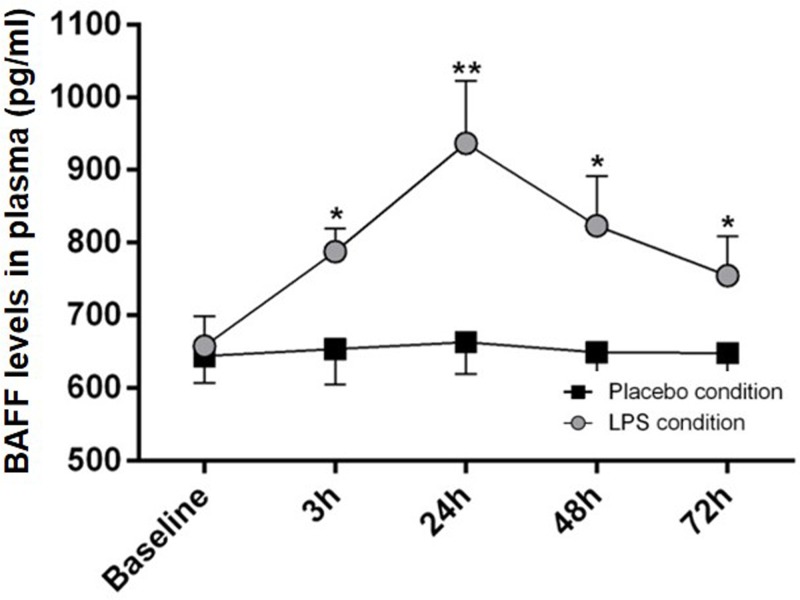
Systemic BAFF levels increase during endotoxemia BAFF levels reached a peak at 24 h after LPS application and did not change after injection of placebo. BAFF levels are given as mean (±SEM) over 72 h; *n*=20. Two-way ANOVA repeated measure analysis was performed followed by post-hoc Bonferroni-corrected paired *t* tests. *P<0.05, **P<0.01, results of post-hoc Bonferroni-corrected *t* tests.

To further elucidate whether the transient changes in the B-cell compartment might be a consequence of an altered cytokine environment, we additionally analyzed BAFF as an important factor for B-cell survival. Endotoxemia led in all subjects to a significant increase in circulating BAFF concentrations (Figure 5). The maximum BAFF concentration was reached at 24 h after LPS injection (F = 19.22, *P*<0.001) and BAFF levels remained significantly elevated until 72 h after LPS challenge. Correlation analysis revealed no significant associations between BAFF serum levels and absolute CD24**^hi^**CD38**^hi^** Breg counts. However, memory B cells showed a negative association with BAFF serum levels at 24 h after LPS challenge (r = −0.534, *P*=0.015). In addition, IL-10 and TNFa serum levels were determined from plasma. Correlation analysis revealed a significant association between IL-10 and plasmablasts (3 h after LPS challenge; absolute plamablast count: r = 0.48, *P*=0.03; relative plasmablast count: r = 0.46, *P*=0.04). Furthermore, TNFa levels were negatively correlated with absolute lymphocyte count (r = −0.55, *P*=0.01). Memory B cells, naïve B cells, CD24**^hi^**CD38**^hi^** Breg and IL-10**^pos^** Breg showed no significant correlation with TNFa or IL-10 plasma levels.

## Discussion

Dysfunction and numerical alterations of the B-cell compartment have been observed in sepsis and most likely contribute to immunoparalysis rendering the host susceptible to secondary infections. In addition to a dysfunction of effector B cells, alterations of the anti-inflammatory Breg population may promote immunoparalysis. Thus, we herein studied the dynamics of the circulating B-cell compartment in a human endotoxemia model and characterized Breg. Our results showed that absolute numbers of B cells, CD24**^hi^**CD38**^hi^**Breg and memory B cells were significantly decreased within 3 h after endotoxin administration. However, the capacity of Breg to produce IL-10 remained unaffected. Interestingly, BAFF serum levels showed a pronounced increase during endotoxemia.

The human endotoxemia model is a common model to study the sepsis-like immune responses and is used as an experimental model for systemic inflammation [18,19,21,24–26]. This model has the advantage over clinical cohort studies that endotoxemia is well controlled and reduces potential bias like duration of antibiotic therapy, type of pathogen and co-morbidities of patients. Hotchkiss et al. [6] demonstrated that patients with sepsis show a severe B-cell deficiency. The authors analyzed spleens derived from septic patients and found a significant decrease in lymphoid follicles and B cells as compared with non-septic patients. Similar findings were reported by several other groups [5,7,27]; in a meta-analysis, diminished numbers of circulating B cells were associated with reduced survival of sepsis patients [28]. Furthermore, Leentjens et al. [25] demonstrated a transient reduction in circulating B cells in a human endotoxemia model after administration of LPS (2 ng/kg body weight). Thus, B-cell deficiency observed in our human model is consistent with data from previous clinical and experimental studies. In our model, B-cell numbers dropped quickly—already 3 h after endotoxin challenge and normalized after 24 h. In patients with sepsis, B-cell deficiency seems to persist during the treatment period presumably due to continuous endotoxemia [7,27]. Despite the total reduction in B cells, the B cell compartment as a whole was skewed with a significant reduction in memory B cells and a proportional increase in mature naïve B cells. This skewing toward the mature naïve B cell compartment has been demonstrated in one other study on septic patients and is likely to contribute to functional immunodeficiency in sepsis [27]. In contrast, Gustave et al. [5] reported no alterations in the B cell memory to naïve ratio. The mechanisms which led to the profound alterations of B cells have not been unraveled. Hotchkiss et al. [6] and Shankar-Hari et al. [27] provide some evidence that especially memory B cells undergo apoptosis in patients with sepsis. This might be a consequence of persistent or over-shooting activation of B cells during sepsis. Indeed, memory B cells are more susceptible to activation-induced cell death than naïve B cells [29]. Acosta-Rodriguez [30] studied the susceptibility of B cells to Fas-mediated cell death in a murine model. B cells exposed to LPS and BAFF were more sensitive to Fas-mediated cell death than B cells cultured in absence of BAFF. These experiments attribute an important role to BAFF in shaping the B cell compartment. We found in our model a sharp increase in BAFF serum levels after LPS challenge paralleling the partial B cell depletion. Therefore, BAFF might be one of the factors promoting B cell deficiency although it promotes B-cell maturation under physiological circumstances. However, redistribution of circulating B cells into peripheral tissues is an alternative explanation for the drop in B cell numbers and the skewing of the B cell compartment.

We also assessed Breg to characterize the anti-inflammatory B cell response possibly contributing to immune dysfunction. Total numbers of CD24**^hi^**CD38**^hi^** Breg, defined according to Blair et al. [10], dropped at 3 h after LPS challenge. Importantly, the fraction of CD24**^hi^**CD38**^hi^** Breg relative to the total B-cell population was stable and not altered. In cohort studies with septic patients, data on CD24**^hi^**CD38**^hi^** Breg are conflicting. In neonatal sepsis patients, the fraction of CD24**^hi^**CD38**^hi^** Breg is reported to be increased whereas in a recent study on adult patients with sepsis the proportion of these cells was not altered [27,31]. In addition, we determined the capacity of B cells to produce IL-10 reflecting Breg function. The fraction of IL-10 producing Breg was stable over time and no difference was observed between LPS and placebo conditions. Thus, endotoxemia did not enhance the anti-inflammatory capacity of Breg. However, it is well known that the efficacy of an anti-inflammatory response is also dependent on the ratio of effector to regulatory cells [32]. If pro-inflammatory effector cells are selectively depleted, regulatory cells are more efficient to suppress the remaining effectors. Therefore, the selective depletion of memory B cells and—as has been previously reported for pro-inflammatory T cells [4,20,21]—may enhance the efficacy of the anti-inflammatory, suppressive function of Breg on the remaining effector cells.

In conclusion, we demonstrated that endotoxemia induces profound alterations of the B cell compartment and leads to partial depletion of memory B cells while the function of the Breg compartment is preserved.

## Perspectives

- Sepsis causes an immune dysfunction of the host increasing the risk for secondary infections; the exact mechanisms leading to immune dysfunction are unknown.
- Endotoxemia leads to profound alterations of the B cell compartment including depletion of memory B cells and Breg.
- Restoration of B-cell homeostasis might be a future therapeutic strategy to treat sepsis-induced immune dysfunction.

## Supporting information

**Supplementary Figure S1:** 

## Abbreviations

ANOVA: analysis of variance
APC: allophycocyanin
BAFF: B-cell activating factor
BAFF-R: B-cell activating factor Receptor
BMI: body mass index
Breg: regulatory B cell
IL: interleukin
LPS: lipopolysaccharide
NEAA: non-essential amino acid
PB: peripheral blood
PBMC: PB mononuclear cells
PDL-1: Programmed Death Receptor Ligand-1
PE-CY7: Phycoerythrin-Cyanin-7
PMA: Phorbol 12-myristate 13-acetate
SEM: standard error of the mean
Teff: effector T cells
