# Supplementary figures and images for: B-cell dynamics during experimental endotoxemia in humans
Source: Biosci Rep. 2019 May 17;39(5):BSR20182347. doi: 10.1042/BSR20182347 (PMC6522728; doi:10.1042/BSR20182347)

# Supplemental Figure S1

**A**

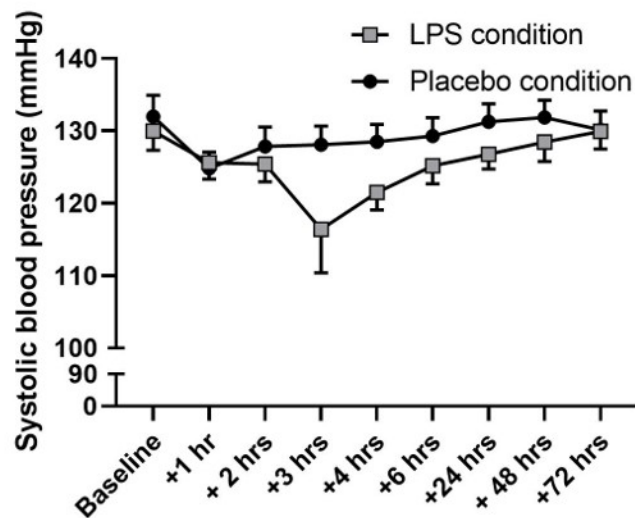

**B**

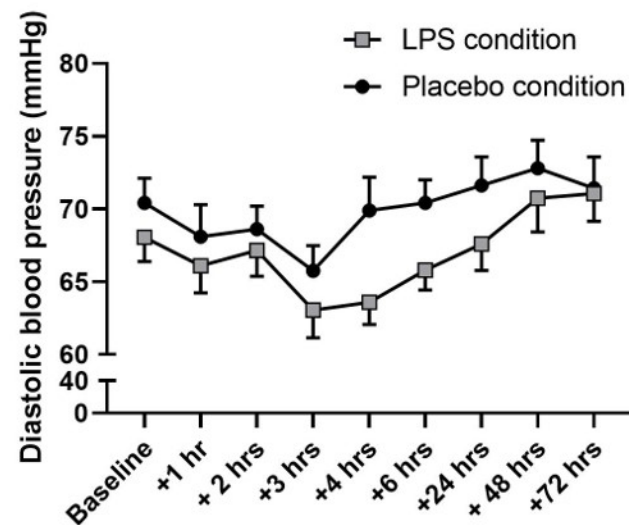

**C**

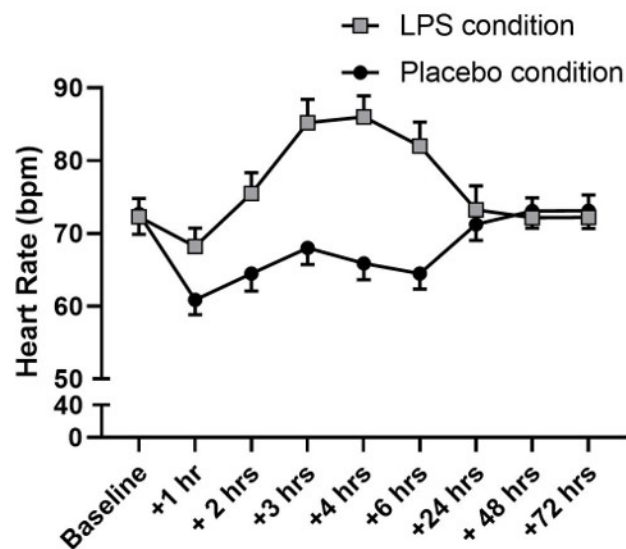

**D**

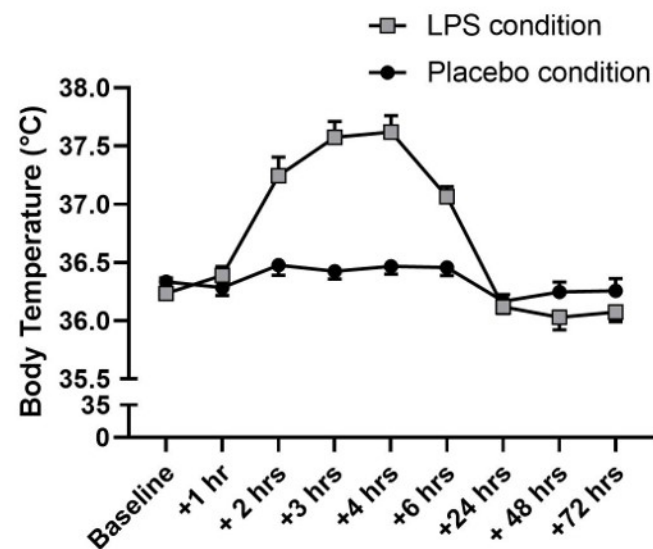

Supplement: Supplementary file 1 [file bsr20182347_Supp1.pdf]
